# Recent advances in rapid multiplex detection of nucleic acid markers using RPA and CRISPR-Cas

**DOI:** 10.3389/fmicb.2026.1810544

**Published:** 2026-05-07

**Authors:** Xiaoping Li, Yuchang Huang, Xinling Zhang, Lailing Du, Yuting Qiu, Lei Jiang

**Affiliations:** 1 Key Laboratory of Artificial Organs and Computational Medicine in Zhejiang Province, Shulan International Medical College, Zhejiang Shuren University, Hangzhou, Zhejiang, China; 2 College of Urban Construction, Zhejiang Shuren University, Hangzhou, Zhejiang, China; 3 Centre of Laboratory Medicine, Zhejiang Provincial People’s Hospital, People’s Hospital of Hangzhou Medical College, Hangzhou, Zhejiang, China

**Keywords:** foodborne pathogens, isothermal amplification, multiplex detection, point-of-care testing, RPA-CRISPR

## Abstract

The integration of recombinase polymerase amplification (RPA) with CRISPR-Cas systems has emerged as a powerful platform for rapid multiplex nucleic acid detection. Compared with quantitative polymerase chain reaction (qPCR) and Next-generation sequencing (NGS), RPA-CRISPR operates isothermally (37 °C–42 °C), requires minimal equipment, and achieves attomolar sensitivity in 20–90 min via collateral cleavage. Recent multiplex strategies, namely two-tube, spatial separation one-tube, and homogeneous one-pot, they have overcome crosstalk and enabled highly multiplexed detection in complex food matrices such as poultry, milk, and lettuce. These approaches are particularly suited for foodborne pathogen screening (e.g., *Salmonella*, *Listeria*), antimicrobial resistance profiling, and on-site surveillance, aligning with the scope of research at the frontier of food microbiology diagnostics. Despite advances, challenges persist in standardization, matrix inhibition, and regulatory approval. This mini-review summarizes recent advances (2020–2025) in RPA-CRISPR multiplex detection, outlines future directions for clinical implementation and food safety deployment, and provides guidance for subsequent research on its practical applications in these fields.

## 1 Introduction

Rapid and reliable detection of microbial pathogens remains a cornerstone challenge in modern food safety surveillance and applied microbiology, underpinning global efforts to mitigate the staggering burden of foodborne diseases. The World Health Organization (WHO) estimates that 93.8 million cases of foodborne illness occur annually worldwide, resulting in 155,00 deaths and economic losses exceeding $110 billion, which costs stemming from medical treatment, product recalls, trade disruptions, and erosion of consumer confidence (Ackerman et al., 2020; Gootenberg et al., 2018; Ayuti et al., 2024). These outbreaks are not merely episodic crises but systemic threats: vulnerable populations (children, the elderly, immunocompromised individuals) face elevated risks of severe complications, including sepsis, organ failure, and death. Among the pathogens most frequently implicated in foodborne outbreaks are *Salmonella enterica* (responsible for ~1.20 million infections per year in the U.S. alone), *Listeria monocytogenes* (with a 20%–30% case fatality rate), *Vibrio parahaemolyticus* (linked to raw seafood consumption), and pathogenic *Escherichia coli* (*E. coli*) strains (e.g., O157:H7), which causes hemolytic uremic syndrome (Li et al., 2020). Their ability to contaminate diverse food matrices such as poultry, dairy, seafood, fresh produce, which persists through processing underscores the urgency of developing detection tools that match the speed of global food supply chains.

The globalization of agriculture and trade has transformed food safety into a transnational imperative. A contaminated batch of spinach grown in California can reach supermarket shelves in Tokyo within 48 h; poultry processed in Brazil may be consumed in Europe the same week. This velocity renders traditional surveillance methods obsolete. Delayed detection allows localized contamination to escalate into multistate or multinational outbreaks, as seen in the 2022 international outbreak of monophasic *Salmonella Typhimurium* linked to chocolate products manufactured in Belgium, which affected multiple countries across Europe and North America (Laisnez et al., 2025). Effective surveillance thus demands tools that deliver results in hours, not days, enabling stakeholders to intercept hazards before they reach consumers.

For decades, culture-based methods reigned as the “gold standard” for pathogen identification. These workflows rely on selective enrichment (to amplify low-level pathogens), isolation on differential media (to distinguish species), and biochemical/serological confirmation steps that collectively take 3–7 days (Xia et al., 2024). While cultures provide isolate characterization (critical for outbreak source tracking), their latency is incompatible with real-time decision-making. A processor cannot hold a shipment for a week while awaiting results, nor can investigators trace an outbreak’s origin if detection lags behind distribution.

Molecular diagnostics emerged as a faster alternative, with quantitative polymerase chain reaction (qPCR) becoming a regulatory mainstay. qPCR detects pathogen-specific nucleic acids with sensitivity down to 1.1 copies per reaction and turnaround times of 90 min (Harrington et al., 2018). However, its reliance on thermocyclers (for denaturation, annealing, extension) and controlled laboratory environments (to prevent contamination) restricts use to centralized facilities. For on-farm testing, border inspections, or small-scale processors, qPCR is impractical. A farmer cannot transport samples to a lab and receive results before a harvest spoils, nor can a border agent deploy a thermocycler in a remote checkpoint.

Next,-generation sequencing (NGS) revolutionized microbial genomics by enabling metagenomic analysis. It is simultaneous identification of pathogens, virulence factors, and antimicrobial resistance (AMR) genes from complex samples (Samantray et al., 2023). Yet, NGS is cost-prohibitive ($250–7,700 per sample) and computationally intensive, requiring bioinformatics expertise to interpret terabytes of data. It is ill-suited for routine screening of thousands of food samples, where throughput and simplicity are paramount (Mirza et al., 2024).

Isothermal nucleic acid amplification technologies address the portability gap left by PCR and NGS. Unlike PCR, these methods operate at constant temperatures (no thermal cycling), enabling use with simple heat blocks or even body heat. Recombinase polymerase amplification (RPA) stands out for its rapid kinetic reactions, which can complete in 10–20 min at 37 °C–42 °C, with minimal equipment (Lobato & O'Sullivan, 2018). Mechanistically, RPA leverages three core components: first, recombinase proteins (e.g., UvsX in *E. coli*) that form complexes with primers and scan double-stranded DNA (dsDNA) for homologous sequences; second, single-strand binding proteins (SSBs) that stabilize displaced DNA strands during strand invasion; third, strand-displacing polymerases (e.g., Bsu polymerase) that extend primers, driving exponential amplification (Wang et al., 2026; Liu et al., 2026). This elegance makes RPA ideal for field use: a battery-powered heater and a reaction tube suffice.

However, RPA alone lacks the specificity needed for multiplex detection (identifying multiple pathogens in one sample). Non-specific amplification, in which primers latch onto unintended sequences, can lead to false positives, particularly when several primer pairs are vying for action in the same reaction. Here, CRISPR-Cas systems provide a transformative solution (Li et al., 2023). Initially characterized as bacterial adaptive immunity (targeting viral DNA), CRISPR nucleases (e.g., Cas12, Cas13) use guide RNAs (gRNAs) to recognize complementary nucleic acids with single-nucleotide precision (Zhao et al., 2024). A landmark discovery was their collateral cleavage activity: upon binding target DNA/RNA, Cas12/Cas13 indiscriminately cleave nearby single-stranded molecules (ssDNA/ssRNA) (Xiao et al., 2025). By incorporating fluorophore-quencher or colorimetric-labeled reporters (e.g., ssDNA oligonucleotides conjugated to FAM-BHQ1), this activity converts target recognition into a measurable signal, forming the basis of platforms like DETECTR (DNA detection) and SHERLOCK (RNA detection) (Shang et al., 2023; Yang et al., 2025).

Integrating RPA with CRISPR creates a synergistic workflow: RPA amplifies target nucleic acids isothermally, and CRISPR validates specificity via gRNA-guided cleavage and reporter activation. This union solves two critical problems: RPA’s speed and portability, and CRISPR’s unmatched specificity. Recent studies report limits of detection (LODs) as low as 10 copies per reaction, with total assay times <60 min, making RPA-CRISPR ideal for field-deployable surveillance (Liu et al., 2021).

Food matrices are rarely contaminated by a single pathogen. Cross-contamination during slaughter (e.g., *Salmonella* from poultry transferring to beef), processing (e.g., *Listeria* spreading via shared equipment), or storage (e.g., *Vibrio* proliferating in warm seafood) means a single sample may harbor multiple hazards (Gonzales-Barron et al., 2023). Conventional multiplex assays (e.g., multiplex PCR) struggle with primer interference (competition for reagents) and signal overlap (inability to distinguish targets), limiting scalability. CRISPR-based systems excel here: orthogonal nucleases (Cas12a, Cas12b, Cas13a) recognize distinct gRNAs, and spectrally distinct reporters (e.g., FAM for *Salmonella*, HEX for *Listeria*) or spatial compartmentalization (microfluidics) eliminate crosstalk (Ipeleng et al., 2025; Liu et al., 2024). For example, a 2023 study developed a multiplex RPA-CRISPR platform that simultaneously detects *Salmonella enterica*, *Listeria monocytogenes*, and *Vibrio parahaemolyticus* in seafood, with LODs of 3.1 × 10^4^, 3.5 × 10^3,^ and 3.9 × 10^2^ cfu/g, respectively (Ndraha et al., 2023).

Another advantage of RPA-CRISPR is tolerance to food matrix inhibitors. Complex samples (e.g., milk, meat, leafy greens) contain lipids, proteins, polysaccharides, and phenolics that inhibit PCR enzymes (e.g., Taq polymerase) by denaturing proteins or sequestering Mg^2+^. RPA polymerases, like Bsu, are naturally tougher: they keep working even with 10% fat or 5 mg/mL protein around, which lets you skip fancy sample prep and just do things like direct lysis without purifying the nucleic acids (Quek et al., 2025; Xie et al., 2024; Wu et al., 2025).

Given these attributes, multiplex RPA-CRISPR is poised to replace legacy methods as the next-generation tool for food microbiology. This paper explores engineering strategies to optimize multiplex performance, applications across food matrices, and future directions to overcome remaining barriers. It can provide guidance for subsequent research on its practical applications in these fields.

## 2 Engineering strategies for multiplex RPA-CRISPR detection

**Figure 1:**
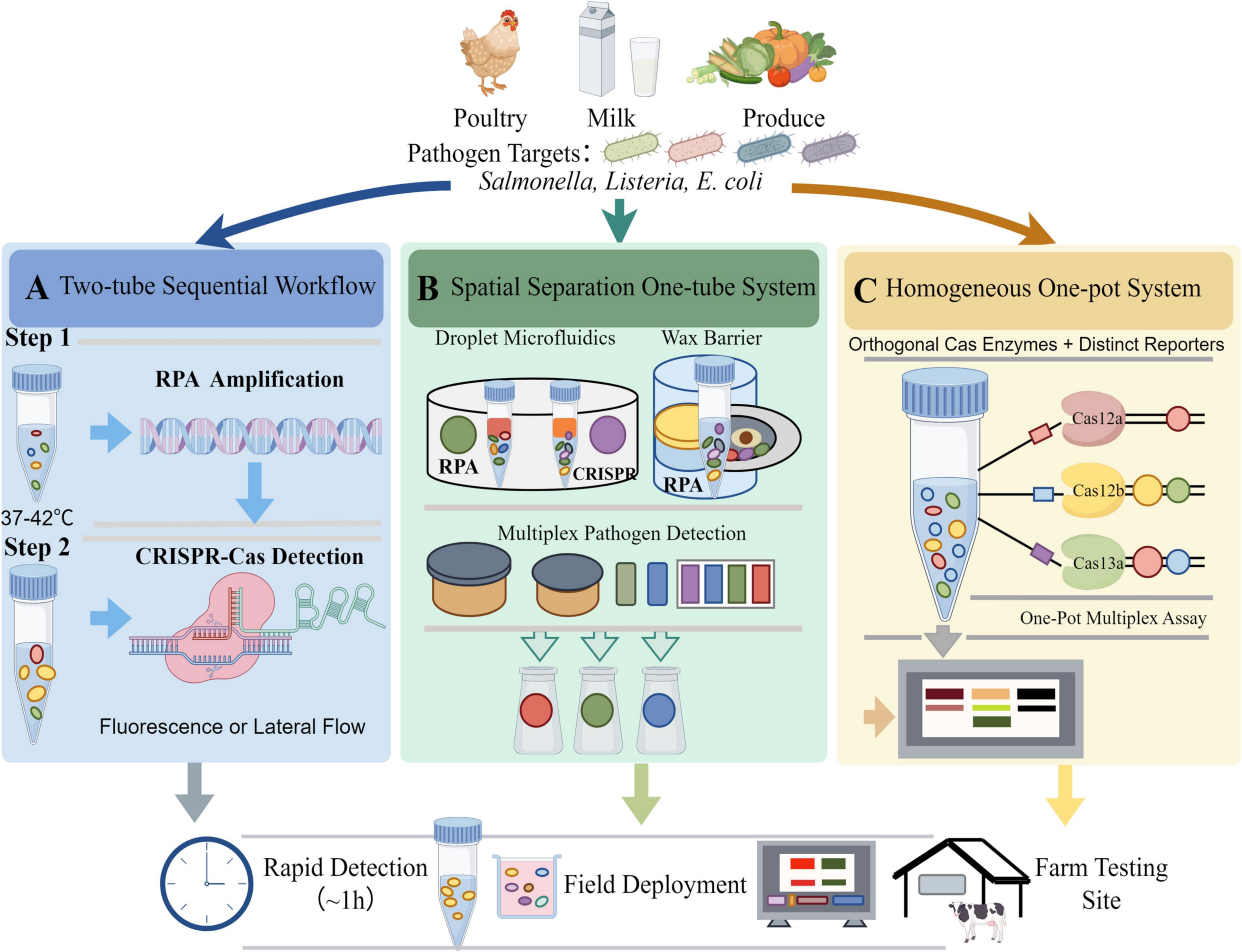
Schematic of multiplex RPA–CRISPR workflows. **(A)** Two-tube sequential workflow in which recombinase polymerase amplification (RPA) and CRISPR-Cas detection are performed in separate reaction vessels to minimize enzymatic interference. **(B)** Spatial separation one-tube systems that compartmentalize amplification and detection reactions using droplet microfluidics, wax barriers, or centrifugal cartridges, enabling multiplex pathogen detection within a closed device. **(C)** Homogeneous one-pot multiplex systems based on orthogonal Cas enzymes (Cas12a, Cas12b, and Cas13a) and distinct reporters, allowing simultaneous multi-target detection in a single reaction mixture. These strategies support rapid detection of foodborne pathogens from complex food matrices such as poultry, milk, and fresh produce, and can be integrated into portable platforms for field deployment. This figure was drawn by Figdraw.

Multiplex RPA-CRISPR assays face three interrelated challenges: first, amplification competition, where multiple primer pairs vie for limited recombinase/polymerase, reducing efficiency for low-abundance targets; second, primer interference, where primers form dimers or bind off-target sequences, increasing false positives; third, reporter crosstalk, architectures, each balancing integration, complexity, and performance (Figure 1). Table 1 provides a comprehensive comparison of the advantages and disadvantages associated with these three RPA-CRISPR multiplex detection strategies.

**Table 1:** Comparison of three RPA-CRISPR multiplex detection strategies.

Strategy	Design principle	Cas protein	Limits of detection (LODs)	Time (min)	Advantages	Challenges	References
Two-tube	Sequential RPA then CRISPR (Two separate tubes)	Cas13a	1 copy/μL (*Salmonella*)	45	Low crosstalk, high specificity	Aerosol contamination risk	An et al. (2021)
Spatial separation one-tube	Centrifugal microfluidic chip (physical isolation)	Cas12a	1 CFU/mL (*Salmonella*)	50	Closed system, zero interference, visual readout	Cost-prohibitive	Chen et al. (2025)
Homogeneous mixing one-tube	All reagents mixed + orthogonal Cas (one sealed tube)	Cas12b	14.4 copies per reaction (*Salmonella*)	60	Contamination-free, simple POCT	UV activation required	Gong et al. (2024b)

### 2.1 Two-tube sequential workflow

The two-tube strategy segregates RPA amplification and CRISPR detection into separate vessels (Figure 1A). In the first tube, RPA amplifies all target nucleic acids simultaneously under optimized conditions (e.g., 39 °C for 15 min). The amplified product is then transferred to a second tube containing CRISPR reagents (Cas nuclease, gRNAs, reporters) and incubated (e.g., 37 °C for 10 min) to trigger cleavage. Researchers have developed a two-step RPA-Cas13a method that enables highly specific detection of *Salmonella* about 45 min, achieving a LODs of 1 copy (An et al., 2021).

Independent optimization: Each step can be optimized independently to achieve peak performance. For instance, RPA parameters such as primer concentration and buffer composition are adjusted to maximize amplification yield. In contrast, CRISPR parameters, including Mg^2+^ concentration and gRNA length, are refined to ensure high specificity. This approach prevents any compromise between the two processes (Zhu et al., 2023).

Reduced inhibitor interference: Food matrices typically inhibit CRISPR enzymes more potently than RPA polymerases. This disparity arises primarily from the larger size of CRISPR proteins and their more stringent cofactor requirements. Separation of the steps enables RPA to occur in crude lysates, whereas CRISPR detection proceeds in a purified environment (e.g., following brief centrifugation) (Monteiro Belo Dos Santos et al., 2026). This keeps crosstalk to a minimum, since CRISPR will not fire up too early during RPA, letting you sidestep any unwanted reporter snips before amplification finishes (Shigemori et al., 2023).

Automation addresses the primary limitation of two-tube workflows, namely contamination arising from manual sample transfers. Microfluidic platforms, such as centrifugal disks and finger-actuated pumps, achieve automated sample routing through the incorporation of fluid control elements that enable stepwise processing and examination of food specimens in a sealed environment, thereby supporting accurate identification of contaminants. Portable variants for field applications rely on passive fluid dynamics, including capillary forces, to forgo mechanical components, thus facilitating deployment in resource-constrained environments (Liu et al., 2024).

Two-tube workflows necessitate precise pipetting and transfer steps, which increase hands-on time (5–10 min per sample) relative to one-pot systems. These workflows also carry a risk of amplicon carryover contamination when transfer tools are not decontaminated, although this can be mitigated through UV sterilization or the use of disposable tips.

### 2.2 Spatial separation one-tube systems

Spatial separation retains amplification and detection in a single device but uses physical barriers (microfluidic compartments, centrifugal cartridges) to isolate reactions (Figure 1B). For example, a centrifugal cartridge may have a “reaction channel” (CRISPR), and a “detection window” (optical readout), with hydrophobic valves controlling fluid flow (Hu et al., 2022).

During centrifugation, RPA reagents mix in the sample chamber, and amplified product is forced into the reaction channel via capillary action or centrifugal force. Valves open sequentially to introduce CRISPR reagents, ensuring amplification precedes detection. Spatial isolation prevents CRISPR nucleases from accessing RPA primers and reporters during amplification, while keeping all steps in one device.

This architecture balances integration and specificity. A 2024 study used a *E. coli* in milk. The chip achieved LODs of 1.2–2.5 CFU/mL, with 95% concordance to culture methods, and reduced assay time to 80 min (vs. 2 h for two-tube) (Wang et al., 2024).

Adding compartments lets you spot more targets without bulking up the device, like a 96-channel chip for high-throughput screening, which boosts scalability. Skipping manual transfers cuts down on contamination by wiping out amplicon carryover. These devices often come sealed and disposable, working with handheld readers such as fluorescence scanners, making them great for field use (Chen et al., 2025).

Microfabrication increases the cost, and valve timing must be precise to avoid premature mixing. However, advances in soft lithography (e.g., PDMS molding) have reduced fabrication costs by 70% since 2020 (Wu et al., 2025).

### 2.3 Homogeneous one-pot systems

One-pot assays perform RPA and CRISPR in a single reaction mixture (Figure 1C), maximizing integration and minimizing handling. All reagents (primers, recombinase, polymerase, Cas nuclease, gRNAs, reporters) are combined at the start, and amplification/detection occur sequentially (RPA first, then CRISPR activation).

Orthogonal nucleases like Cas12a (targets DNA, cuts ssDNA reporters), Cas12b (targets DNA, distinct PAM needs), and Cas13a (targets RNA, cuts ssRNA reporters) help dodge cross-reactivity. Take Cas12a, for instance. It binds target DNA with a 5′-TTTV PAM next to the spacer sequence. Cas13a, on the other hand, lacks a PAM requirement but may favor certain 3′ PFS motifs on the RNA target. This ensures that each nuclease activates exclusively in response to its designated target (Buddhachat et al., 2021; Huang et al., 2022).

RPA works best around 39 °C, while Cas12 and Cas13 kick in effectively at 37 °C. With a gradual temperature increase of about 1 °C per minute from 25 °C to 39 °C, RPA finishes up before CRISPR activates, avoiding early reporter cleavage. In reporter design, link distinct fluorophores like FAM (excited at 495 nm), HEX (535 nm), and Cy5 (650 nm) to ssDNA reporters for concurrent multi-channel fluorescence readout.

Optimized one-pot reactions hold up well against two-tube methods in sensitivity. A 2024 study detecting *Salmonella* in chicken samples reached 14.4 copies per reaction with 98% specificity, yielding results in 50 min (Gong et al., 2024a). Another detected four pathogens in lettuce at 1 CFU/mL, matching qPCR closely (Boukharouba et al., 2022). By skipping transfers, the method becomes straightforward for those with little lab background, such as field inspectors or farmers. Costs drop too, since a single tube lowers consumable use and cuts per-test expenses by around 40%. It scales nicely to 96-well plates for efficient batch testing in the lab.

Amplification competition poses a hurdle, as a strong primer set in high amounts can crowd out others, limiting detection of rarer targets. Adjusting ratios, perhaps 1:1:1 for three targets, or choosing primers with comparable melting points can balance it out (Wu et al., 2024). Reporter crosstalk is another concern, with activated Cas12a potentially cutting HEX-tagged reporters meant for Cas13a. Options to fix this involve enzyme-targeted blockers, such as anti-Cas13a antibodies, or strengthening reporters through robust changes like phosphorothioate bonds (Shu et al., 2025).

## 3 Applications in food safety and clinical diagnostics

Multiple RPA-CRISPR platforms have undergone validation in a range of food matrices, encompassing poultry, dairy products, and fresh produce, with further applications in clinical diagnostics, including the identification of foodborne pathogens in patient specimens. Herein, we outline principal performance indicators and highlight key applications metrics for their deployment in food safety and clinical settings.

### 3.1 Detection of major foodborne pathogens

Poultry constitutes a major reservoir for *Salmonella* and *Campylobacter* species, with reports suggesting that as much as 41% of retail chicken meat harbors *Salmonella* in the United States. A study published in 2025 introduced a multiplex recombinase polymerase amplification-CRISPR assay for the identification of *Salmonella enterica serovar Indiana* and *Listeria monocytogenes* in chicken breast tissue. This approach incorporated a single-pot reaction scheme employing Cas12a for *Salmonella* detection and Cas13a for *Listeria* detection, alongside lateral flow strips for optical result assessment. The method permitted swift identification of artificially contaminated specimens, displaying superior sensitivity and specificity over quantitative PCR, stemming from the resilience of recombinase polymerase amplification to suppressive agents in avian adipose tissue (Aljuwayd et al., 2023).

Raw milk and cheese represent high-risk matrices for *Listeria monocytogenes*—which can proliferate at refrigeration temperatures—and *Escherichia coli* O157:H7. In a recent study, a spatially separated microfluidic chip was employed to detect these pathogens in pasteurized milk. Hydrophobic valves on the chip ensured that recombinase polymerase amplification (RPA) occurred prior to CRISPR-based detection, while the inclusion of MnO₂ nanosheets in the RPA mixture adsorbed inhibitory proteins, thereby enhancing amplification efficiency. The method was capable of detecting pathogens at levels compliant with EU regulatory limits, i.e., 100 CFU/g for *L. monocytogenes* in ready-to-eat foods (D'Ambrosio et al., 2026).

Food products are susceptible to *Listeria monocytogenes* contamination from various environmental and processing sources. A study developed an LM-RPA-Cas12a-LFA assay with Cas12a to detect the pathogen in complex food matrices. The assay incorporated aptamer-functionalized magnetic beads to enrich targets, reducing LODs to 1.35 CFU/mL in complex food matrices, demonstrating excellent reproducibility and stability. This approach holds promise for food safety by enabling swift on-site screening of samples to avert contamination-related recalls and public health threats. In clinical diagnostics, it could streamline identification of bacterial agents in patient specimens, supporting prompt management of gastrointestinal illnesses (Wang et al., 2026).

### 3.2 Monitoring antimicrobial resistance genes

AMR is a growing threat in foodborne pathogens: the WHO lists *blaNDM-1* (carbapenem resistance) and *mcr-1* (colistin resistance) as critical priorities. Multiplex RPA-CRISPR enables simultaneous detection of pathogens and AMR genes, providing actionable data for surveillance.

A 2024 study developed a one-pot assay for *Salmonella* and *blaNDM-1*/*mcr-1* in pork. The assay used Cas12a (for *Salmonella*) and two Cas13a enzymes (for *blaNDM-1* and *mcr-1*), with Cy5-labeled reporters for *mcr-1* and FAM for *blaNDM-1*. Spiked samples were detected with 96.64% sensitivity for both genes. This platform could help regulators track AMR spread from livestock to humans, it is critical for implementing “One Health” surveillance (Gong et al., 2024b; Balaga et al., 2024).

### 3.3 Portable and point-of-care platforms

Field deployment requires miniaturization and user-friendliness. Researchers have integrated RPA-CRISPR with handheld fluorescence detectors: A 2025 study used a palm-sized device to detect *Salmonella* in chicken feces. The device reads fluorescence via fiber optics, with results displayed on a touchscreen (Samantray et al., 2023).

Portable platforms for point-of-care testing facilitate “sample in, answer out” analysis of pathogens in diverse matrices. One such system employs centrifugal microfluidics to combine magnetic bead nucleic acid extraction, recombinase-assisted amplification, and CRISPR/Cas13a detection within a single chip, enabling multiplex identification of 10 viruses in under 45 min with detection thresholds reaching 1–5 copies per reaction across plasmid, plasma, swab, and blood specimens. Equipped with preloaded reagents, active rotation, and programmable wax valves, the device demands minimal user input and no specialized training. In food safety, it offers potential for expeditious field-based screening of contaminated commodities to mitigate viral outbreaks linked to supply chains. For clinical diagnostics, it supports swift evaluation of patient samples, enhancing response to infectious diseases in underserved regions (Zhang et al., 2024).

Take electrochemical sensors, for instance. Microfluidic electrochemical RPA-CRISPR biosensors make it possible to quickly, sensitively, and simultaneously pick up foodborne pathogens like *Salmonella* and *Staphylococcus aureus* even in messy matrices. These platforms integrate sample processing, RPA amplification, and Cas12a-based recognition with a nanomaterial-modified electrode, achieving the LODs as low as 3 CFU/mL and detection times within 65 min, offering a practical tool for on-site food safety monitoring (Guo et al., 2025).

## 4 Discussion

While RPA-CRISPR assays hold great promise for rapid foodborne pathogen detection, they face notable hurdles in multiplexing, matrix interference, and regulatory hurdles. This discussion delves into these technical challenges, validation requirements, and future innovations to boost sensitivity, scalability, and real-world applicability.

### 4.1 Technical challenges

In multiplex RPA, primers targeting highly abundant sequences (e.g., *Salmonella* in poultry) can outcompete primers for low-abundance targets (e.g., *Listeria*), thereby reducing detection sensitivity. To address this challenge, primer concentrations can be optimized using mathematical models such as Michaelis–Menten kinetics, which allow adjustment of primer ratios based on the relative abundance of each target (Yao et al., 2024). Alternatively, Cas-assisted amplification co-expresses Cas nucleases with RPA, enabling selective degradation of off-target amplicons; however, this approach introduces additional experimental complexity (Yin et al., 2023).

Activated Cas12a can inadvertently cleave reporters intended for Cas13a targets, particularly when the reporters share similar sequences. Strategies to mitigate such off-target activity include the co-addition of nuclease-specific inhibitors, such as small molecules like EDTA, to suppress unintended Cas12a cleavage (Cordon-Obras et al., 2022). Another approach involves designing sequence-specific reporters with deliberate mismatches to non-target nucleases; for example, a FAM-labeled reporter carrying a 3′ mismatch at Cas13a’s cleavage site remains intact when exposed to off-target nucleases (Cordon-Obras et al., 2022).

High concentrations of lipids, proteins, and phenolic compounds can still inhibit RPA-CRISPR reactions. Advanced sample preparation strategies have been developed to mitigate these effects. For instance, nanomaterial-based enhancers such as MnO₂ nanosheets and graphene oxide have been employed, with MnO₂ adsorbing proteins and lipids, and graphene oxide removing phenolic compounds (Xia et al., 2024). Additionally, coating magnetic beads with specific aptamers enables selective pathogen capture, thereby both concentrating target nucleic acids and removing inhibitory substances from the sample (Wang et al., 2026).

### 4.2 Regulatory validation

Diagnostic methods must demonstrate reproducibility (consistent results across labs) and robustness (performance in diverse matrices) to gain regulatory approval. International frameworks like ISO 16140 (validation of alternative methods) and Association of Official Analytical Collaboration (AOAC) International official methods require: For each category under evaluation, a minimum of 60 individual samples should be tested, comprising at least three distinct types with a minimum of 20 representative samples per type (i.e., 3 types × 20 samples = 60 samples). Fractional positive results should be observed for each type by either the reference or alternative method, ensuring that the samples are not uniformly positive or negative. Ideally, approximately 50% of samples per type (i.e., 10 positive and 10 negative) should yield a positive result, although a range of 25%–75% is considered acceptable. Across each category, at least 30 samples should test positive by either the reference or alternative method.

Few RPA-CRISPR assays have completed full validation: several recent studies validated *Vibrio* assays in multiple seafood matrices (e.g., fish, shrimp, clams), but most platforms remain in the research phase and have not yet met formal ISO 16140 certification (Messelhäusser et al., 2010).

### 4.3 Future directions

New CRISPR nucleases with improved orthogonality will expand multiplex capacity. For example, Cas14 (smaller than Cas12, targets ssDNA) and Cas12f (ultracompact, no PAM requirement) enable smaller reaction volumes and more targets per assay (Harrington et al., 2018; Wu et al., 2023). Moreover, Machine learning (ML) is increasingly used to optimize CRISPR guide RNA design. Deep learning models trained on large-scale Cas13 guide datasets can accurately predict on-target activity and off-target tolerance, outperforming earlier heuristic design methods and improving the specificity of CRISPR-based RNA targeting systems (Silverstein et al., 2025).

Lab-on-a-chip devices will automate “sample-to-answer” workflows: a 2024 platform integrated sample lysis, RPA, CRISPR, and detection in a single chip, reducing hands-on time to <30 min (Fozouni et al., 2021). Furthermore, ML algorithms will simplify data interpretation, especially for complex multiplex signals. A 2025 study trained a convolutional neural network (CNN) to analyze LFS images from a 2-plex assay, achieving 96.5% accuracy in classifying positive/negative bands (Xue et al., 2025). ML can also correct for matrix effects (e.g., distinguishing true positives from inhibitor-induced fluorescence).

Future diagnostic chips are expected to integrate fully automated sample-in answer-out functionality, allowing users to simply introduce a food homogenate while the device performs nucleic acid extraction, amplification, and CRISPR detection without manual intervention. In parallel, ongoing developments in biosensor integration are likely to enable true multi-analyte detection within a single assay. Such platforms could simultaneously screen for foodborne pathogens, antimicrobial resistance genes, and toxin markers such as aflatoxin, thereby providing a more comprehensive assessment of food safety in a single analytical run (Guo et al., 2025).

## 5 Conclusion

Multiplex RPA-CRISPR systems represent a paradigm shift in food microbiology: they combine the speed and portability of isothermal amplification with the specificity of CRISPR, enabling rapid, sensitive, and multiplex detection of foodborne pathogens in complex matrices. Engineering technology innovations create two-tube workflows, spatial separation, and homogeneous one-pot systems, which have addressed key challenges of amplification competition, primer interference, and reporter crosstalk, while applications across poultry, dairy, seafood, and produce demonstrate real-world utility (Fang et al., 2026).

Critical barriers persist in technical domains, including amplification competition, matrix effects, regulatory aspects, validation and practical factors, like cost. However, progress in enzyme engineering, microfluidics, and machine learning stands ready to surmount these hurdles. With appropriate validation and regulatory approval, multiplex RPA-CRISPR could become the backbone of global food safety surveillance, enabling early detection of outbreaks, targeted recalls, and proactive protection of public health (Zhou et al., 2024). As the food supply chain becomes increasingly complex, these tools will ensure that rapid detection aligns with the speed of rapid distribution, which serves as a key requirement for a safer and more resilient food system and clinical pathway implementation (Xie et al., 2024; Li et al., 2024). Ultimately, it can provide important monitoring and protection for implementing “One Health.”
